# Demographics of COVID-19 hospitalisations and related fatality risk patterns

**DOI:** 10.1016/j.healthpol.2022.07.005

**Published:** 2022-10

**Authors:** Daniela Ghio, Simona Bignami-Van Assche, Nikolaos I. Stilianakis

**Affiliations:** a European Commission, Joint Research Centre (JRC), Ispra, Italy; b Department of Demography, Université de Montréal, Montréal, Canada; c Department of Biometry and Epidemiology, University of Erlangen-Nuremberg, Erlangen, Germany

**Keywords:** COVID-19 Hospitalisations, COVID-19 Hospitalisation Fatality Risk, COVID-19 Demographic Risk Factors

## Abstract

The assessment of hospitalisations and intensive care is crucial for planning health care resources needed over the course of the coronavirus disease 2019 (COVID-19) pandemic. Nonetheless, comparative empirical assessments of COVID-19 hospitalisations and related fatality risk patterns on a large scale are lacking.

This paper exploits anonymised, individual-level data on SARS-CoV-2 confirmed infections collected and harmonized by the European Centre for Disease Prevention and Control to profile the demographics of COVID-19 hospitalised patients across nine European countries during the first pandemic wave (February – June 2020). We estimate the role of demographic factors for the risk of in-hospital **mortality**, and present a case study exploring individuals’ comorbidities based on a subset of COVID-19 hospitalised patients available from the Dutch health system.

We find that hospitalisation rates are highest among individuals with confirmed SARS-CoV-2 infection who are not only older than 70 years, but also 50-69 years. The latter group has a longer median time between COVID-19 symptoms’ onset and hospitalisation than those aged 70+ years. Men have higher hospitalisation rates than women at all ages, and particularly above age 50. Consistently, men aged 50-59 years have a probability of hospitalisation almost double than women do. Although the gender imbalance in hospitalisation remains above age 70, the gap between men and women narrows at older ages. Comorbidities play a key role in explaining selection effects of COVID-19 confirmed positive cases requiring hospitalisation.

Our study contributes to the evaluation of the COVID-19 burden on the demand of health-care during emergency phases. Assessing intensity and timing dimensions of hospital admissions, our findings allow for a better understanding of COVID-19 severe outcomes. Results point to the need of suitable calibrations of epidemiological projections and (re)planning of health services, enhancing preparedness to deal with infectious disease outbreaks.

## Highlights

• Understanding demographics of COVID-19 hospitalisations is crucial for health policy.
• In-hospital mortality provides a baseline to assess COVID-19 management effectiveness.
• COVID-19 hospitalisations vary more over age and gender than across countries.
• COVID-19 hospitalisations of 50-69 age-group individuals should not be overlooked.
• Women are generally less likely to be hospitalised than men are.

## 1 Introduction

The new coronavirus disease 2019 (COVID-19), first identified in Wuhan, China in December 2019, has heavily impacted health care systems around the world, since severe cases require hospitalisation, including intensive care [1]. Hospital-based studies have found that age, sex, and individuals’ pre-existing medical conditions are strongly associated with an increased risk of mortality among patients infected with severe acute respiratory syndrome coronavirus 2 (SARS-CoV-2) [2], [3], [4], [5], [6], [7]. Nonetheless, the demographic characteristics of individuals hospitalised because of COVID-19 and related fatality patterns have rarely been studied at the population level, especially in comparative perspective [8], [9], [10], [11], [12]. On the contrary, most international cross-country studies have focused on COVID-19 fatality burden [13], notably excess mortality [14].

There is growing consensus that one of the main reasons why early epidemiological models of COVID-19 [15] have failed in their predictions of needed hospital capacity is that they were based on limited data and did not fully take into account demographic risk factors and individuals’ comorbidities [16]. Currently, more reliable and comparable evidence on the demographics and severe outcomes is available for the first wave of COVID-19 pandemic in Europe. This requires to be assessed in order to recalibrate projections and appropriately plan health care resources while vaccine programmes remain burdened with policy challenges [17]. In this paper, we address these needs by exploiting anonymised, individual-level data collected and harmonised by the European Centre for Disease Prevention and Control (ECDC)_to compare the demographics of COVID-19 hospitalisations and related fatality risk patterns across nine European countries (Austria, Germany, Finland, Italy, Latvia, Luxembourg, the Netherlands, Norway, and Portugal) during the first wave of the COVID-19 pandemic.

### 1.1 DATA

The ECDC collects harmonized data on COVID-19 from European Union (EU) Member States^1^ and European Economic Area (EEA) countries. This data collection refers to anonymised information about individuals with laboratory-confirmed (defined as positive real-time reverse transcription–polymerase reaction chain) SARS-CoV-2 infections, that EU and EEA countries are requested to report to ECDC within 24 hours from identification.

The analyses carried out in this paper are based on subsets from this ECDC data collection, which we were given access through the European Surveillance System (TESSy) operated by the ECDC [18] from February to June 2020. We exclude from the analysis countries that, as of June 2020, had reported to ECDC individual-level information on less than 70 percent 2 of official infections and/or deaths: Belgium, Cyprus, Denmark, Estonia, Greece, Hungary, Poland, Lithuania, Slovakia and Romania. Additionally, the following countries could not be included: Ireland, because gender information of individuals infected with SARS-CoV-2 was not reported to ECDC; and Czechia, Iceland, Malta and Sweden, because they provided information on hospitalisations for less than 40 percent of confirmed SARS-CoV-2 infections. Thus, our analysis focuses on almost one million SARS-CoV-2 infections in nine countries: Austria, Germany, Finland, Italy, Latvia, Luxembourg, the Netherlands, Norway, and Portugal (Table 1). The COVID-19 pandemic has unfolded at different times in these countries. Based on the ECDC data collection, Italy and Germany recorded their first cases in late February 2020, followed by countries in Northern Europe, whereas sustained community transmission in Eastern Europe started in the third week of March. Policy response mainly consisted of population based measures (lockdowns were first imposed in Italy on March 11 2020, and most European countries followed suit shortlly afterwards) to limit the spread of the infection and the number of fatalities associated with the disease^3^. Lockdown measures were effective to curb the spread of COVID-19 and were gradually lifted in mid-May 2020 in most countries [19]. Our analysis, which refers to all confirmed infections between February 1 and June 30, therefore captures the first pandemic wave in Europe.

**Table 1:** Number of SARS-CoV-2 infections confirmed between February 1 and June 30, 2020, percent of individuals with confirmed SARS-CoV-2 infection who were hospitalised, percent of individuals with confirmed SARS-CoV-2 infection with unknown hospitalisation status, and percent of individuals with confirmed SARS-CoV-2 infection who were hospitalised that required admission to intensive care, by sex and country.

Country	Number of individuals with confirmed SARS-CoV-2 infection			Percent of individuals with confirmed SARS-CoV-2 infection who were hospitalised			Percent of individuals with confirmed SARS-CoV-2 infection who were hospitalised and required admission to intensive care *			Percent of individuals with confirmed SARS-CoV-2 infection with unknown hospitalisation status		
	Men	Women	Total	Men	Women	Total	Men	Women	Total	Men	Women	Total
Austria	18525	17147	35672	6	5	6	14	9	12	6	6	6
Finland	4610	4463	9073	11	11	11	64	62	63	33	29	31
Germany	126052	128413	254465	14	11	13	8	16	12	17	16	17
Italy	128851	145794	274645	40	25	32	12	12	12	0	0	0
Latvia	669	607	1276	17	14	16	7	13	10	0	0	0
Luxembourg	2990	2771	5761	10	8	9	21	27	24	0	0	0
Netherlands^1^	28835	42033	70868	26	11	17	n/a	n/a	n/a	27	24	25
Norway	4804	4697	9501	13	10	12	3	4	3	9	9	9
Portugal^1^	26160	32496	58656	12	10	11	14	10	12	12	12	12
All countries	341496	378421	719917	25	17	21	11	13	12	17	19	19

1 Information on intensive care is not available for the Netherlands.

⁎ Among individuals with confirmed SARS-CoV-2 infection who were hospitalised, proportion who required admission to intensive care. *Source*: TESSy data (see Data & Methods).

For each confirmed SARS-CoV-2 infection, the TESSy anonymised database includes the following information: the patient's age and gender; hospitalisation status (yes, no, or unknown); utilisation of intensive care (with and without mechanical ventilation) when hospitalised (yes, no); and clinical outcome (dead, alive, or still in treatment). Table 1 presents descriptive statistics for the countries included in the analysis. The percentage of confirmed SARS-CoV-2 infections with missing information on hospitalisation status varies from six percent in Austria to 31 percent in Finland. Information on intensive care is available for all hospitalised cases, but not available for the Netherlands. Because the end of the chosen period of analysis corresponds to the end of the first pandemic wave in Europe, less than 1% of SARS-CoV-2 infections are censored (still in treatment). For approximately half of hospitalised COVID-19 cases in Germany, the Netherlands, and Portugal, we also had access to both the date of COVID-19 symptoms’ onset and the date of hospitalisation. Finally, for a subset of SARS-CoV-2 infections confirmed in the Netherlands between February 1 and May 7, 2020 (N=8,727), we had access to information on the patient's pre-existing medical conditions (cancer, diabetes, cardiac disorder, kidney-related conditions and renal diseases, and lung disease).

## 2 Methods

The analysis consists of two parts. In the first part, we describe the demographics of COVID-19 hospitalisations using three main indicators: A) age- and sex-specific rates of hospitalisation and intensive care, B) age- and sex-adjusted probabilities of hospitalisation, and C) age- and sex-specific median times from COVID-19 symptoms’ onset to hospitalisation.

A Age- and sex-specific rates of hospitalisation and intensive care are calculated, separately for men and women, as the proportion of individuals in each age group^4^ who were hospitalised/admitted to ICU given confirmed SARS-CoV-2 infection. For each country, rates of hospitalisation and intensive care per 100,000 population are presented and compared through the tool of age pyramids (for the Netherlands, only hospitalisation rates are provided);
B Age- and sex- adjusted probabilities of hospitalisation among confirmed SARS-CoV-2 infections are derived from the multivariable logistic regression model using country fixed effects to control for unobserved contextual characteristics such as national testing strategy for SARS-CoV-2. Results are graphed separately for each country;
C Age- and sex-specific median times from COVID-19 symptoms’ onset to hospitalisation, as estimated by survival analysis carried out separately for men and women. The analysis is restricted to the only three countries with available data: Germany, the Netherlands, and Portugal.

In the second part of the analysis, we estimate in-hospital mortality risk, or the risk of mortality among confirmed SARS-CoV-2 infections who required hospitalisation, using multivariate logistic regression modelling with country fixed effects. As indicated earlier, less than 1% of SARS-CoV-2 infections in our database are censored (excluded from the analysis) because referring to patients that are still in treatment as of June 2020. In the case of the Netherlands, we also estimate in-hospital mortality risk controlling for patients’ pre-existing medical conditions. We apply multivariate Cox proportional hazard models, separately for men and women, to a subset of COVID-19 hospitalised patients from February 1 to May 7, 2020. We include a time interaction with each co-morbidity as a way of testing the proportional-hazards assumption.

All analyses are performed using Stata statistical software, version 15.

## 3 Results

### 3.1 Demographics of COVID-19 hospitalisations

#### 3.1.1 Age- and sex-specific rates of hospitalisation and intensive care

**Fig. 1:**
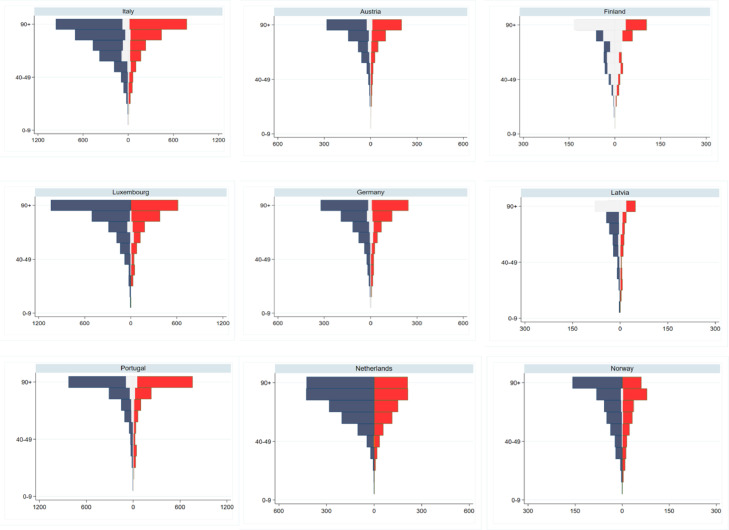
Age- and sex-specific hospitalisation and intensive care rates (per 100,000 population) among SARS-CoV-2 infections confirmed between February 1 and June 30, 2020 in Austria, Finland, Germany, Italy, Latvia, Luxembourg, Norway, and Portugal. blue = males; red = females; white = intensive care *Note:* For the Netherlands, age and sex-specific intensive care rates are not available.

*Source*: our calculations from TESSy data (see Data & Methods).

Hospitalisation rates in the nine countries included in the analysis (see Appendix A, Table A2) are highest in Italy (177 per 100,000 for men and 120 per 100 000 for women), and lowest in Latvia (13 per 100,000 for men and 8 per 100,000 for women). Fig. 1 shows that, across age groups, hospitalisation rates are highest for individuals older than 70 years, consistently with the existing literature [9], [10], [11], [12], [13]. Adults aged 50–69 years are the second largest group of SARS-CoV-2 positive individuals who required hospitalisation, especially men. In this group, hospitalisation rates are at least two times higher than among 20–29 years old. Among men and women aged 50–59, the highest rates of hospitalisation are recorded in Italy (187 and 103 per 100,000 respectively), while the lowest are recorded in: Latvia (22 and 10 per 100,000 for men and women respectively); Austria (26 and 16 per 100,000 for men and women respectively); Finland (33 and 26 per 100,000 for men and women, respectively).

As media reports of overwhelmed ICUs in Italy and New York City circled the globe in early March 2020, the need for intensive care has been one of the most important issues during the first wave of the COVID-19 pandemic [20]. In Fig. 1, we thus compare the rates of admission to intensive care (with and without invasive ventilation) across the countries included in the analysis.

The rates of admission to intensive care among the selected countries (see Appendix A, Table A2) are highest in Italy (20 per 100,000 for men and 20 per 100,000 for women), and lowest in Norway (1 per 100,000 both for men and women). In Austria, Finland, Italy, Latvia and Portugal, the male rates of admission to intensive care are higher than the female ones.

The rates of admission to intensive care vary by age in Finland, in Italy after 50+ and in Latvia and Portugal among elderly; in the other countries, the age-specific variability is less evident.

**Fig. A1:**
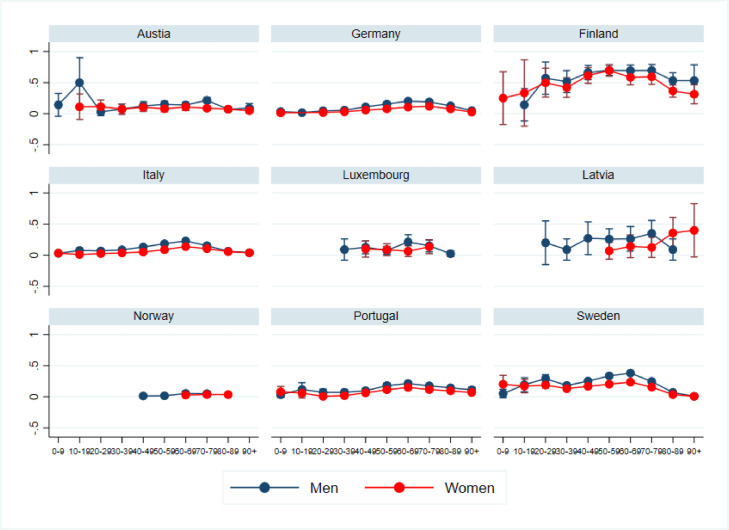
Age and sex-adjusted probability of ICU admission among COVID-19 cases who were hospitalised between February 1 and June 30, 2020, in Austria, Finland, Germany, Italy, Latvia, Luxembourg, Norway, and Portugal

*Source*: our calculations from TESSy data (see Data & Methods).

Exploring the probability of ICU admission, 40-69 age-group hospitalised men are more likely to be admitted to intensive care than hospitalised women in the same age group, while the probability at other age groups are quite similar (see Appendix A, Fig. A1).

Although the presence of confounding factors (notably co-morbidities) requires further investigation, the observed disparities in the ICU utilisation reflect the combination of a different expression of COVID-19 severity by age and gender, with the ICU availability and access. Gender disparities in utilisation of intensive care seem to be only partially explained by differences in the age of patients. For instance, existing studies show that fewer care home residents are admitted to ICU for COVID-19 than younger individuals with confirmed SARS-CoV-2 infection [21]. A possible explanation consists in the use of non-invasive respiratory support (such as continuous positive airway pressure or high-flow nasal oxygen therapy) that are frequently provided outside ICU, but it is well understood that treatment choice is a complex and individualised decision based on risk-benefit assessments at local level, involving patients, families, carers and health professionals [22].

Another striking result from Fig. 1 is that intensive care has represented a small part of hospitalisations in all countries considered, except for Finland^5^. When we compare the percentage of patients admitted to ICU with the number of hospitalised patients, this is around 12% in Austria, Germany^6^, Italy and Portugal, and for the nine countries as whole (see Table 1). This value is much lower than the one assumed by early epidemiological models [15], which estimated that 30 out 100 hospitalised COVID-19 cases would have required intensive care.

### 3.2 Age- and sex-adjusted probabilities of hospitalisation among confirmed SARS-CoV-2 infections

**Fig. 2:**
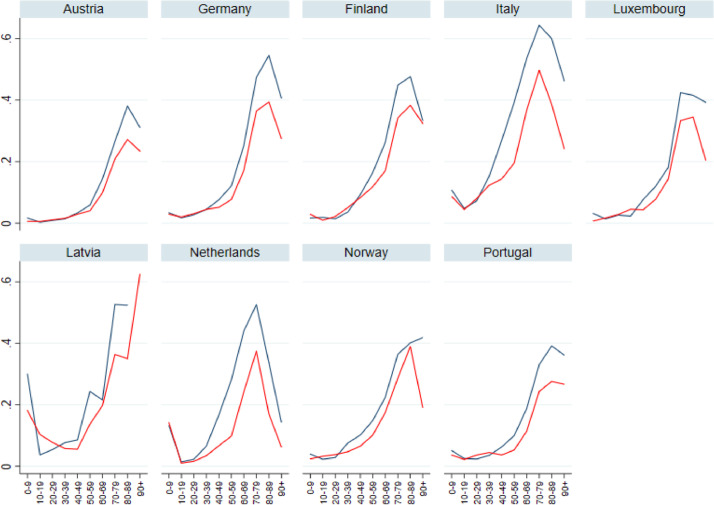
Age- and sex-adjusted probability of hospitalisation among SARS-CoV-2 infections confirmed between February 1 and June 30, 2020 in Austria, Finland, Germany, Italy, Latvia, Luxembourg, the Netherlands, Norway, and Portugal blue = males; red = females

*Source*: our calculations from TESSy data (see Data & Methods).

Except for Latvia, the probability of hospitalisation increases with age and peaks at age 70-79 years (see Fig. 2). At this age, the probability of hospitalisation is highest in Italy (for men .66–95% CI [.652–.662], for women .53–95% CI [.522–.533]) and Germany (for men .50–95% CI [.493–.506], for women .37 - 95% CI [.358–.370]). Consistently with Fig. 1, men have a higher probability of hospitalisation than women do at ages 40-79. For instance, in Germany men aged 50–59 years have a probability of hospitalisation (.21–95% CI [.206–.214]) almost double than the corresponding figure for women (.10–95% CI [.100–.105]). The gender imbalance in hospitalisation tends to narrow at older ages (80+).

### 3.3 Age- and sex-specific median times from COVID-19 symptoms’ onset to hospitalisation

The probabilities of hospitalisation in Fig. 2 do not take into account the length of time elapsed between COVID-19 symptoms’ onset and hospital admission. Using available datasets for Germany, the Netherlands and Portugal, we verify whether the median time between symptoms’ onset and hospital admission varies by age and sex (see Table 2). We find that this is indeed the case. Like the probability of hospitalisation, the median number of days between symptoms’ onset and hospitalisation increases with age. However, it is the longest for confirmed SARS-CoV-2 infections between age 30 and 69 years, and it declines at older ages. Considering that, as mentioned earlier, in these age groups we find the second largest group that has required hospitalisation because of COVID-19 (see Fig. 1), this finding is an additional reason why hospitalisation needs of prime age adults should not be overlooked in the planning of healthcare resources for dealing with the pandemic. Contrary to the probability of hospitalisation, there are small sex differences in the median time of hospitalisation, which is approximately one day higher for men and women at almost all ages. Overall, the median time between symptoms’ onset and hospital admission varies between 4 and 7 days across the countries included in the analysis.

**Table 2:** Median time (in days) between the date of symptons’ onset and hospitalisation, for Germany, the Netherlands and Portugal, among SARS-CoV-2 infections confirmed between February 1 and June 30, 2020, by age and sex.

Age group	MEN			WOMEN		
	Germany	Netherlands	Portugal	Germany	Netherlands	Portugal
<30	5	6	3	4	5	3
30-59	7	8	6	7	8	5
60-69	7	8	6	6	8	6
70-79	5	7	5	5	7	4
80+	4	6	3	4	5	3
All cases	6	7	5	6	7	4

*Source*: our calculations from TESSy data (see Data & Methods).

## 4 Fatality risk patterns related to hospitalisations

Probabilities of in-hospital mortality given SARS-CoV-2 infection across age and gender groups are presented in Table 3. In-hospital mortality increases linearly with age above 50 for both men and women, with the highest probability of dying for confirmed SARS-CoV-2 infections requiring hospitalisation found among those aged 80+.

**Table 3:** a. Age- and sex-adjusted probability of in-hospital mortality given SARS-CoV-2 infection confirmed between February 1 and June 30, 2020 in Austria, Finland, Germany, Italy, Latvia, Luxembourg, the Netherlands, Norway, and Portugal (standard error in parentheses): Men.

Age group	Austria	Germany	Finland	Italy	Luxembourg	Latvia	Netherlands	Norway	Portugal
0-9	0.001	0.001	0.001	0.001	0.000	0.000	0.001	0.001	0.000
	(0.000)	(0.000)	(0.001)	(0.001)	(0.000)	(0.000)	(0.001)	(0.000)	(0.000)
10-19	0.000	0.000	0.000	0.000	0.000	0.000	0.001	0.000	0.000
	(0.000)	(0.000)	(0.000)	(0.000)	(0.000)	(0.000)	(0.000)	(0.000)	(0.000)
20-29	0.002***	0.002***	0.003***	0.003***	0.001***	0.001***	0.003***	0.002***	0.001***
	(0.000)	(0.000)	(0.001)	(0.001)	(0.000)	(0.000)	(0.001)	(0.000)	(0.000)
30-39	0.004***	0.004***	0.007***	0.007***	0.003***	0.003***	0.008***	0.005***	0.002***
	(0.000)	(0.000)	(0.001)	(0.001)	(0.000)	(0.001)	(0.001)	(0.001)	(0.000)
40-49	0.013***	0.013***	0.022***	0.023***	0.009***	0.011***	0.024***	0.015***	0.006***
	(0.001)	(0.001)	(0.002)	(0.001)	(0.001)	(0.002)	(0.001)	(0.001)	(0.000)
50-59	0.036***	0.037***	0.063***	0.064***	0.026***	0.031***	0.068***	0.044***	0.017***
	(0.002)	(0.001)	(0.004)	(0.002)	(0.003)	(0.006)	(0.002)	(0.003)	(0.001)
60-69	0.108***	0.111***	0.178***	0.181***	0.079***	0.094***	0.189***	0.127***	0.053***
	(0.004)	(0.002)	(0.010)	(0.003)	(0.008)	(0.017)	(0.003)	(0.008)	(0.002)
70-79	0.266***	0.272***	0.395***	0.399***	0.206***	0.237***	0.413***	0.305***	0.144***
	(0.008)	(0.003)	(0.016)	(0.003)	(0.018)	(0.036)	(0.005)	(0.015)	(0.004)
80-89	0.453***	0.460***	0.598***	0.602***	0.371***	0.414***	0.615***	0.500***	0.276***
	(0.010)	(0.004)	(0.016)	(0.003)	(0.026)	(0.048)	(0.005)	(0.017)	(0.007)
90+	0.577***	0.584***	0.710***	0.714***	0.493***	0.539***	0.725***	0.623***	0.387***
	(0.011)	(0.006)	(0.014)	(0.005)	(0.028)	(0.049)	(0.006)	(0.017)	(0.009)
**b. Age- and sex-adjusted probability of in-hospital mortality given SARS-CoV-2 infection confirmed between February 1 and June 30, 2020 in Austria, Finland, Germany, Italy, Latvia, Luxembourg, the Netherlands, Norway, and Portugal (standard error in parentheses): Women**									

Age group	Austria	Germany	Finland	Italy	Luxembourg	Latvia	Netherlands	Norway	Portugal
0-9	0.001**	0.001**	0.002**	0.002**	0.001*	0.001*	0.002**	0.001**	0.001**
	(0.001)	(0.001)	(0.001)	(0.001)	(0.000)	(0.001)	(0.001)	(0.001)	(0.000)
10-19	0.000	0.000	0.000	0.000	0.000	0.000	0.000	0.000	0.000
	(0.000)	(0.000)	(0.000)	(0.000)	(0.000)	(0.000)	(0.000)	(0.000)	(0.000)
20-29	0.001***	0.001***	0.001***	0.001***	0.000***	0.000***	0.001***	0.001***	0.000***
	(0.000)	(0.000)	(0.000)	(0.000)	(0.000)	(0.000)	(0.000)	(0.000)	(0.000)
30-39	0.002***	0.002***	0.004***	0.004***	0.001***	0.002***	0.004***	0.002***	0.001***
	(0.000)	(0.000)	(0.001)	(0.001)	(0.000)	(0.000)	(0.001)	(0.000)	(0.000)
40-49	0.005***	0.005***	0.010***	0.010***	0.004***	0.005***	0.010***	0.006***	0.002***
	(0.000)	(0.000)	(0.001)	(0.001)	(0.001)	(0.001)	(0.001)	(0.001)	(0.000)
50-59	0.013***	0.013***	0.023***	0.023***	0.009***	0.011***	0.024***	0.015***	0.006***
	(0.001)	(0.001)	(0.002)	(0.001)	(0.001)	(0.002)	(0.001)	(0.001)	(0.000)
60-69	0.057***	0.058***	0.097***	0.099***	0.041***	0.049***	0.104***	0.068***	0.027***
	(0.003)	(0.002)	(0.006)	(0.002)	(0.004)	(0.009)	(0.003)	(0.005)	(0.001)
70-79	0.175***	0.179***	0.276***	0.279***	0.131***	0.153***	0.291***	0.204***	0.089***
	(0.006)	(0.003)	(0.013)	(0.003)	(0.013)	(0.026)	(0.005)	(0.011)	(0.003)
80-89	0.306***	0.312***	0.442***	0.447***	0.239***	0.274***	0.461***	0.348***	0.169***
	(0.009)	(0.004)	(0.016)	(0.003)	(0.020)	(0.039)	(0.005)	(0.016)	(0.005)
90+	0.411***	0.418***	0.556***	0.560***	0.332***	0.374***	0.574***	0.457***	0.243***
	(0.010)	(0.005)	(0.017)	(0.004)	(0.024)	(0.046)	(0.005)	(0.017)	(0.006)

*** statistically significant at 1% level, ** statistically significant at 5% level.

*Source*: our calculations from TESSy data (see Data & Methods).

Our estimates quantify the heightened mortality risk of men age 50+ who required hospitalisation in all countries included in the analysis. This shows that an important factor of observed mortality differentials by gender is the much higher risk of dying of male cases who need to be hospitalised, especially at old ages, compared to women. This may result from socioeconomic factors combined with heightened biological risk [[23], [24]], as well as the selection effect of cases requiring hospitalisation.

To investigate the latter issue, we estimate the risk of in-hospital mortality controlling for the pre-existing medical conditions reported by individuals with confirmed SARS-CoV-2 infection in the Netherlands. Table A1 in Appendix A reports the subsample composition of the examined patients by age, gender and comorbidity. Confirmed SARS-CoV-2 infections who were hospitalised had at least one more comorbidity than those who were not (63% higher probability among hospitalised patients with at least a comorbidity than among hospitalised patients without pre-existing medical conditions, corresponding to proportion .47, 95% CI [.463–.484] and proportion .17, 95% CI [.167–.178] respectively). For women, at all ages the majority of hospitalised cases had lung disease (30–40%) or cardiac disease (approximately 25%). In each age group, hospitalised men were less likely to have cancer and more likely to have diabetes than non-hospitalised men. The reverse was true for women.

**Fig. 3:**
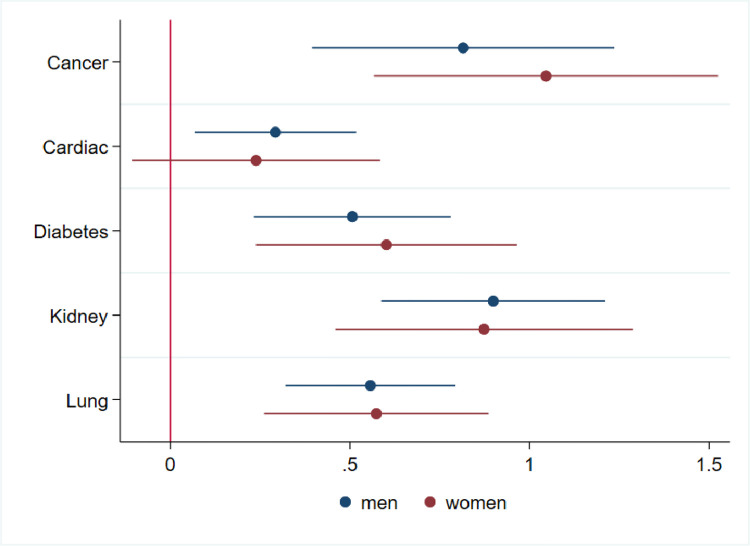
Age- and sex-adjusted hazard ratio (dots) and standard error (line) for in-hospital mortality given SARS-CoV-2 infection confirmed in the Netherlands between February 1 and May 7, 2020, by pre-existing medical condition

*Source*: our calculations from TESSy data (see Data & Methods). Appendix A.

Controlling for individuals’ co-morbidities, we find that cancer, lung disease and diabetes are indeed associated with higher in-hospital mortality risk among women, while chronic heart disease and reduced kidney function increase in-hospital mortality risk among men (see Fig. 3) and Appendix A, Table A4 with the interaction controls)). Testing the proportional-hazards assumption shows that no change in the proportional hazard is significant when checked over the time of hospitalisation among patients affected by co-morbidities. Therefore, the results of event history modelling indicate that the factors associated with mortality risk among SARS-CoV-2 positive cases who were hospitalised are invariant to the length of hospitalisation.

## 5 Discussion

In this paper, we exploit comparable data for nine European countries during the first wave of COVID-19 to carry out a large-scale empirical assessment of the demographic characteristics (age and sex) of confirmed SARS-CoV-2 infections that required hospitalisation, and their associated fatality risk patterns.

Most studies have focused on the concentration of COVID-19-related fatalities among the elderly to assess the burden of COVID-19 on national health care systems. Since early on, this approach has been recognised to overlook morbidity [25], but comparative assessment of COVID-19-related hospitalisations at the individual level are still lacking. Our study thus contributes to the evaluation of the COVID-19 burden on healthcare systems showing that, beyond specific hospital settings, there has been a need for hospitalisation because of COVID-19 across all age groups, especially for men, during the first pandemic wave.

Consistently across countries, we find that men have a higher probability of hospitalisation than women do at all ages. This gender gap in the probability of hospitalisation is particularly large for the working age population between 50 and 69 years, and it narrows above age 70. Bearing in mind that local contextual circumstances and individual factors affect decisions concerning the allocation and use of intensive care therapies, differences by gender are revealed when looking at demographics of ICU utilisations across the countries considered. Yet, gender disparities in utilisation of intensive care seem to be only partially explained by differences in the age of patients.

Furthermore, we provide a set of comparable estimates of in-hospital mortality risk across the selected countries, which represent one of the mostly common used indicators to evaluate effectiveness of clinical management and treatment (including vaccination) for diseases. We find a much higher risk of dying among 50+ years old men who need to be hospitalised compared to women.

Using a subsample of COVID-19 patients residing in the Netherlands, we find that cancer, lung disease and diabetes are associated with higher in-hospital mortality risk among women, while chronic heart disease and reduced kidney function increase in-hospital mortality risk among men.

Our analysis empirically shows to what extent COVID-19 fatality is an inadequate indicator to assess health care utilization for the disease. The number of COVID-19-related deaths is an indicator of the fatality of SARS-CoV-2 infection, which is just the tip of the iceberg of the severity of the disease. Focusing on COVID-19 fatality is a narrow view that excludes a large proportion of SARS-CoV-2 infections requiring health care resources. As the case of the Italian region of Lombardy presented in the Appendix-B indicates, the proportion of COVID-19 hospitalised patients and related fatality patterns have changed over time because of several factors, such as the evolution of the international epidemiological situation, the adopted policy of testing and treatment of infected patients, and the allocation of health care resources (including testing capacity). These dynamics have significant implications when planning hospital capacity because they could lead to an inefficient allocation of resources for facing pandemic waves [26].

By assessing how health care resources have been allocated during the earliest stages of the pandemic, our study gives key-insights into the COVID-19 burden across the selected countries which allow enhancing health care systems preparedness. Moving forward, epidemiological models need to be calibrated based on parameters derived from empirical data like those presented in this study.

Limitations of the study are related to data availability. Despite the ECDC dataset used in our analysis is a unique, harmonised data collection effort among European official providers of epidemiologic statistics, there are a number of limitations that should be highlighted. EU/EEC countries have been reporting to ECDC data on confirmed SARS-CoV-2 infections, but for most of them information on hospitalisation was not enough for completed analyses of the demo-epidemiologic profiles over time (see [18] for further details on ECDC data completeness). As for June 2020, only three countries have provided information on the date of COVID-19 symptoms’ onset and hospital admission, and no information on the date of admission to intensive care unit. Unavailable for all countries is also information on the date of hospital discharge, which would allow for analyses of the duration of hospital stay that are critical to plan the allocation of healthcare resources. Ensuring that these information gaps are filled is an important step in the ongoing fight against COVID-19 pandemic.

## Disclaimer

The information and views set out in this paper are those of the authors and do not necessary state or reflect the official opinion of the European Centre for Disease Prevention and Control (ECDC). Neither the European Union institutions and bodies nor any person acting on their behalf may be held responsible for the use, which may be made of the information contained therein.

The accuracy of the authors’ statistical analysis and the findings they report are not the responsibility of ECDC. ECDC is not responsible for conclusions or opinions drawn from the data provided. ECDC is not responsible for the correctness of the data and for data management, data merging and data collation after provision of the data. ECDC shall not be held liable for improper or incorrect use of the data.

The scientific output expressed does not imply a policy position of the European Commission. Neither the European Commission nor any person acting on behalf of the Commission is responsible for the use that might be made of this publication. For information on the methodology and quality underlying the data used in this publication for which the source is neither Eurostat nor other Commission services, users should contact the authors. The designations employed and the presentation of material do not imply the expression of any opinion whatsoever on the part of the European Union concerning the legal status of any country, territory or of its authorities.

The views expressed in this article are purely those of the authors and may not, under any circumstances, be regarded as an official position of the European Commission.

## Funding

This research did not receive any specific grant from funding agencies in the public, commercial, or not-for-profit sectors.

## Declaration of Competing Interest

None.

## Figures and Tables

**Table A1:** Individuals with SARS-CoV-2 infection confirmed in the Netherlands between February 1 and May 7, 2020 who had a least one comorbidity, percentage who had cancer, cardiac disease, diabetes, kidney disease or lung disease, by age and sex.

	Cancer	Cardiac disease	Diabetes	Kidney disease	Lung disease	
MEN	%	%	%	%	%	Number
0-49	2.7	26.7	24.0	5.3	41.3	150
50-59	6.9	31.4	26.8	7.1	27.8	392
60-69	7.6	35.1	22.4	5.3	29.6	733
70-79	5.8	35.7	21.3	8.3	28.9	1125
80+	4.5	38.4	17.6	10.8	28.7	705
WOMEN						
0-49	8.0	19.3	14.7	7.3	50.0	149
50-59	10.7	25.0	18.0	4.9	41.4	244
60-69	11.8	22.2	20.4	6.2	39.4	406
70-79	9.2	27.4	21.7	8.3	33.4	599
80+	10.2	25.3	21.2	7.5	35.8	493

*Source*: TESSy data (see Data & Methods).

**Table A2:** Rates of hospitalisation and intensive care admission per 100,000 population, median age of population and individuals with confirmed SARS-CoV-2 infection and by hospitalisation status (hospitalised, admitted to intensive care, not hospitalised, with unknown hospitalisation status).

Country		MEN						
	Hospitalisation rate^1^	Rate of ICU admission^1^	Median age (years)					
			All SARS-CoV-2 confirmed infections	Hospitalisation for COVID-19	ICU admission for COVID-19	Unknown hospitalisation status	Not hospitalised cases	Population
Austria	26	4	39	69	70	33	37	42
Germany	42	3	43	68	69	37	40	44
Finland	19	12	38	61	62	32	37	41
Italy	177	20	58	69	65	n/a	50	46
Luxembourg	92	10	41	61	64	n/a	38	39
Latvia	13	4	44	58	60	n/a	41	40
Netherlands^2^	86	n/a	56	68	n/a	43	53	42
Norway	24	1	45	60	66	40	42	39
Portugal	63	9	44	69	64	46	41	44
Country		WOMEN						
	Hospitalisation rate^1^	Rate of ICU admission^2^	Median age (years)					
			All SARS-CoV-2 confirmed infections	Hospitalised COVID-19 cases	COVID-19 cases admitted to ICU	Unknown hospitalisation status	Not hospitalised cases	Population
Austria	21	2	41	74	71	37	40	45
Germany	35	5	46	73	74	41	44	48
Finland	17	10	42	62	60	36	41	45
Italy	120	15	58	73	70	n/a	54	48
Luxembourg	71	13	41	66	61	n/a	39	40
Latvia	8	3	47	63	80	n/a	45	47
Netherlands^2^	54	n/a	52	69	n/a	45	51	44
Norway	18	1	44	62	75	41	41	41
Portugal	57	6	47	75	68	52	45	47

1 Rates per 100,000 population

2 Information on intensive care is not available for the Netherlands. *Source*: our calculations from TESSy data (see Data & Methods). Rates’ denominators are from EUROSTAT [27].

**Table A3:** Age distribution (%)of SARS-CoV-2 confirmed infections with unknown hospitalisation status, by sex and country.

	Austria		Germany		Finland		The Netherlands		Norway	
Age	Women	Men	Women	Men	Women	Men	Women	Men	Women	Men
0-9	5	5	4	4	4	5	1	1	3	3
10-19	14	17	8	9	10	10	8	10	7	7
20-29	20	22	19	22	24	29	20	21	20	19
30-39	16	16	16	18	15	19	13	14	17	18
40-49	16	14	15	15	14	14	14	13	19	18
50-59	14	13	16	16	16	12	16	16	15	16
60-69	6	6	7	8	8	7	8	10	9	10
70-79	5	4	5	4	3	3	6	8	7	6
80-89	3	2	6	3	3	1	8	7	3	2
90+	1	1	3	1	1	0	4	2	1	0

*Source*: our calculations from TESSy data (see Data & Methods).

**Table A4:** Age and sex-adjusted hazard ratio (HR) and standard error for in-hospital mortality given SARS-CoV-2 infection confirmed in the Netherlands between February 1 and May 7, 2020, by pre-existing condition (cancer, cardiac disease, diabetes, kidney disease or lung disease).

	Men HR	Interaction	Women HR	Interaction
**COMORBIDITIES**				
**CANCER**	2.260*** 0.485	1.004 0.020	2.846*** 0.696	0.969 0.020
**CARDIAC**	1.340** 0.154	0.993 0.009	1.269 0.224	1.000 0.016
**DIABETES**	1.660*** 0.232	1.003 0.013	1.825*** 0.338	0.984 0.018
**KIDNEY**	2.458*** 0.391	0.985 0.013	2.395*** 0.506	0.992 0.018
**LUNG**	1.746*** 0.211	0.992 0.011	1.775*** 0.283	0.989 0.014

*** statistically significant at 1% level ** statistically significant at 5% level

Note: *Interaction* controls the continuously time-varying of comorbidities’ hazard risk with respect to the duration of hospitalisation.

*Source*: our calculations from TESSy data (see Data & Methods).

**Fig. B1:**
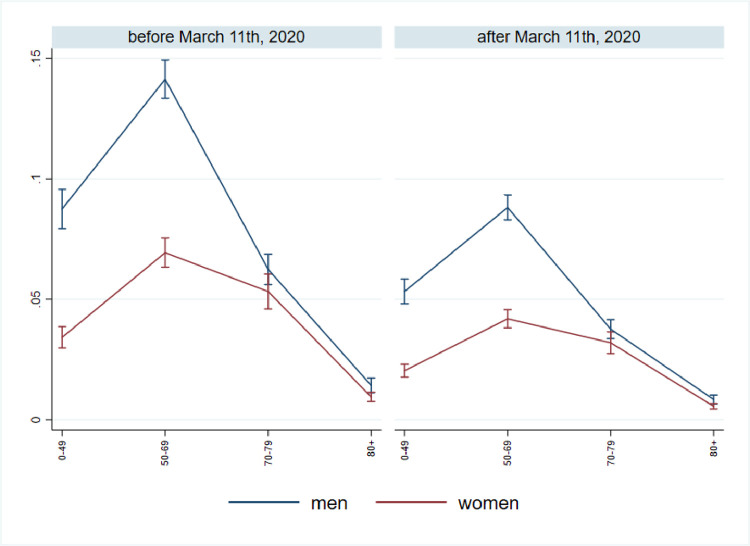
Age- and sex-adjusted probability of hospitalisation, Lombardy region: February 24 -April 25, 2020

*Source*: our calculations from TESSy data

**Fig. B2:**
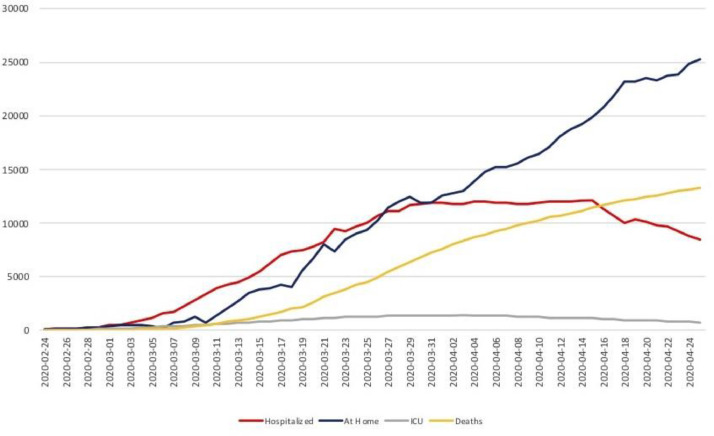
Cumulative number of confirmed SARS-CoV-2 infections in Lombardy between February 24 and April 25, 2020, by status (hospitalised, at home, ICU, died)

*Source*: Department of Civil Protection. Datasets available from https://github.com/pcm-dpc/COVID-19

## Appendix A

Fig. A1 and Tables A1, A2, A3 and A4

## Appendix B The case of the Italian region of lombardy

Italy was the first Western country hit by the COVID-19 pandemic after China, and it has recorded one the deadliest tolls because of the pandemic [28]. The Italian region of Lombardy, where most fatalities occurred during the first wave of COVID-19 [29], is an important case study to show the importance of understanding and monitoring the demographics of COVID-19-related hospitalisations for appropriately planning healthcare resources.

After the identification of first positive cases in the northern towns of Codogno and Vò on February 21^st^, 2020, the Italian government quarantined these municipalities and their surroundings. Yet, the first restrictions introduced in the regions of Lombardy (and Veneto) were ineffective in limiting the spread of the virus. In Lombardy as a whole, the estimated effective reproduction number was under the epidemic threshold for the initial phases of the epidemic, and it then started quickly growing as the virus spread in different geographical areas [30]. Therefore, on March 11^th^, Italy enforced the first national lockdown in Europe [19].

Previous studies have attested that, in this early phase of the Italian pandemic, the transmission of SARS-CoV-2 increased very fast in several municipalities in Lombardy such as Bergamo [[29], [30]]. The demand for ICU could not be met and health care resources became inaccessible [[31], [32]]. Fig. B1 indeed shows that, immediately following the national lockdown, the probability of hospitalisation dropped substantially for all age groups under 70 years.^7^ The probability to be hospitalised among men aged 50–69 dropped from 12% to 7%, and among women from 7% to 4%. At the same time, the probability of hospitalisation for men aged 70–79 years decreased from 5% to 3%, and from 5% to 2 % for women.

As cases were not treated in hospital, especially among 50–69 years old, the number of COVID-19 patients isolated at home dramatically increased (Fig. B2). Until March 11^th^, for every three hospitalised patients, there was one isolated at home. On March 31^st^, for every hospitalised patient, there was one isolated at home. As April 25^th^, for every hospitalised patient, there were three patients isolated at home. As the number of COVID-19 positive cases isolated at home increased, the number of COVID-19-related fatalities increased at the same pace. The mechanisms linking these two trends remain unexplored, although numerous studies and the public attention have highlighted COVID-19-related excess of mortality in the region, which in certain municipalities such as Bergamo topped 500 % [14]. However, quantifying the COVID-19 component of the total excess mortality is made difficult because COVID-19 related fatalities have been largely underestimated in the emergency phase due to the lack in testing, completeness, and delays in administrative reporting, such as it has been observed in other countries [33].

## Footnotes

1 Five of the twenty-seven EU countries (Bulgaria, Croatia, France, Slovenia, Spain) have not reported to ECDC individual-level information on SARS-CoV-2 infections. Regarding the EEA countries, only Iceland and Norway have provided data during the reference period.

2 In the context of cross-sectional time-series and survival data, when informative gaps exist for individuals, national sequences are seen as entities [27]. When comparing different sequences, the limiting-case would converge to zero when all sequences are equal (high concentration). The concentration is settled at 70% being the measure consistent with the overall concentration distribution.

3 The start and end dates of the public response measures and the testing strategies in place in each country are available via the country profiles compiled by ECDC at: https://covid19-country-overviews.ecdc.europa.eu.

5 Table A3 in Appendix A shows that the median age of unknown cases is closer to the median age of not hospitalised cases than to the estimate for hospitalised case. This suggests that unknown missed hospitalisation status’ cases are more likely individuals that were not hospitalised. This would imply that COVID-19 cases with missed hospitalisation status in Finland could not significantly bias estimated hospitalisation rates reported for this country.

6 Our estimates are in line with comparable estimates for Germany [5].

## Footnotes

4 For all countries, population estimates by age and sex were obtained from Eurostat [34].
